# How to Examine a Sick Child

**Published:** 1882-10-20

**Authors:** 


### HOW TO EXAMINE A SICK CHILD.

TRANSLATED BY R. MATÁS, M.D.

Dr. Decroizilles inaugurated his course of pædiatry, at the children's hospital, by indicating the method to be followed in the examination of sick infants for the proper detection of disease.

We summarize:

If we are dealing with the newborn infant great advantage will be derived from an examination during sleep. If proper care is taken not to waken the slumberer, an opportunity is offered to us for the favorable observation of the physiognomy, attitude, respiration and pulse. If awake, we should notice any peculiarity about its cry, the manner in which it takes the breast; we should closely examine its jaws to determine the existence of any abnormalities of conformation, and if the lips are able to suck the examiner's finger well.

After these brief observations are made, the babe should be stripped and all the regions of its body should be successively submitted to inspection.

The normal attitude of the newborn infant is that of flexion, with the head dropped upon the breast; toward the end of the second month the head is maintained elevated; in the fourth or fifth the infant easily sits up; about the eighth or ninth it begins to support itself upon its feet, and, finally, at about the termination of the fourteenth or fifteenth month it attempts to walk.

The cutaneous surface, which is usually very red during the first few days, may assume a yellow icteroid tinge on the third or fourth day. It does not attain its definite color till the fourth month.

To examine the grown up infant is a more difficult matter. It becomes necessary sometimes to recur to forcible measures, but these should always be practiced with gentleness.

The examination in such cases should begin as before, by divesting the little patient of all his garments in order that an exact "inventory" may be taken of his body.

The wrappings which envelope the child should be minutely examined, so that the nature of the alvine discharges and the character of the urinary secretion be properly ascertained. If the diaper is not damp with urine, a slight pressure over the hypogastrium will usually suffice to cause the discharge of a certain quantity of this fluid.

As the infantile pulse is usually very rapid, the thermometer should be used regularly to recognize the presence of fever.

The faces of infants are a matter of great importance in the study and diagnosis of their diseases. The healthy babe's face is usually calm and placid; it becomes contracted and corrugated upon the advent of pain; choleraic disorders will cause a peculiar pinching of the faces and a characteristic drawing down of the oral angles; whilst pneumonia will tinge the cheeks with a circumscribed blush.

Once the existence of fever is recognized, an examination of the throat should never be neglected. To inspect an infant's throat is not always an easy matter, as most practitioners know. The child should be held firmly in the arms of some strong person in order that it may be rendered as passive as possible; the nose should be gently pinched between the thumb and index, in order to force it to open its mouth; then the introduction of the tongue depressor will become an easy measure.

Auscultation should always be practiced directly with the ear over the chest. The vesicular murmur in a child is always more intense than in the adult; and on a level with the great bronchial trunks, over the spine and scapular regions, it is remarkably reinforced, so that it assumes a rude and almost bronchial character.

The heart's sounds are louder and clearer than in the adult. Their maximum intensity is heard only over one place, the third intercostal space, to the left.

In the infant, percussion should succeed auscultation.

The pulmonary resonance extends, posteriorly, to the twelfth dorsal vertebræ; on the right to the tenth and eleventh only; in front and to the left it extends to the fourth or fifth rib, and on the right side to the third only.—*New Orleans Med. and Surg. Jour.*

